# The 48th ASMS Conference on Mass Spectrometry and Allied Topics

**DOI:** 10.1002/1097-0061(200012)17:4<322::AID-YEA46>3.0.CO;2-M

**Published:** 2000

**Authors:** Francesco Brancia

**Affiliations:** Michael Barber Centre for Mass Spectrometry UMIST Sackville Street Manchester M60 1QD UK

## Abstract

The general theme of the meeting was the application of mass spectrometry to pharmaceutical and biotechnological research. The majority of the oral presentations and posters were concerned with the development and application of all mass spectrometric techniques related to proteomics.


**Yeast**
*Yeast* 2000; **17**: 322–326.

**Meeting Review**

### The 48th ASMS conference on mass spectrometry and allied topics http://www.asms.org

**Long Beach Convention Centre, California, USA. 11–15 June 2000.**
**Sponsors: The American Society of Mass Spectrometry**

Francesco Brancia*
*Michael Barber Centre for Mass Spectrometry, UMIST, Sackville Street, Manchester M60 1QD, UK*

*Correspondence to:
F. Brancia, Michael Barber Centre for Mass Spectrometry, UMIST, Sackville Street, Manchester M60 1QD, UK.
E-mail: Francesco.brancia@stud.umist.ac.uk*

Received: 13 September 2000
Accepted: 15 September 2000

### Abstract

**The general theme of the meeting was the application of mass spectrometry to pharmaceutical and biotechnological research. The majority of the oral presentations and posters were concerned with the development and application of all mass spectrometric techniques related to proteomics. Copyright © 2000 John Wiley & Sons, Ltd.**

### Background

Matrix-assisted laser desorption ionization (MALDI) and electrospray ionization (ESI) represent the two complementary techniques used to achieve an unambiguous identification of protein mixtures: MALDI is an high-throughput technique, well suited to rapid identification of dozens of gel-separated proteins. The protein is hydrolysed by a proteolytic enzyme (trypsin, lysine C), generating mixtures of peptides that are analysed in the mass spectrometer. The comparison of high accuracy MALDI peptide mass fingerprinting against a sequence database provides the most likely protein hits. Recently, it has emerged that MALDI peptide mapping alone may not allow identification of proteins, due to differential detection of peptide moieties and to the high degree of redundancy of present genomes. Therefore, combining MALDI peptide mapping with nanoelectrospray tandem mass spectrometry has been suggested. Unlike the previous ionization technique, ESI is labour-intensive and time-consuming, but it provides additional structural information through fragmentation of the selected ion in the mass analyser: the signals resulting from collisional induced dissociation are used to establish the partial sequence of the selected peptide. Furthermore, ESI is readily coupled to several separation techniques, allowing fractionation of complex protein mixtures by eluting each component at different retention times.

### Oral presentations

Over the last 10 years, extensive effort has been made to apply the combination of the matrix-assisted laser desorption ionization technique with time-of-flight mass spectrometry to Sanger sequencing mixtures for the purpose of rapid DNA sequencing. Electrospray ionization, however, has not been extensively explored for the purpose of DNA sequencing, due in part to limitations associated with electrospray of mixtures. New methods that require electrospray ionization Fourier transform ion cyclotron resonance mass spectrometry (ESI–FT–ICR) have been reported to accurately characterize PCR products. Of particular interest was the presentation entitled '**Broad-based Genotyping of Short Tandem Repeat Loci Using**

Copyright © 2000 John Wiley & Sons, Ltd.

**ESI–FT–ICR Mass Spectrometry'**, in which James C. Hannis (Commonwealth University, Richmond, VI, USA) described the analysis of short tandem repeats (STRs) or microsatellites, (stretches of repeating DNA sequences with remakable variability found dispersed through the entire human genome) using ESI–FT–ICR. Sequencing oligonucleotides through mass spectrometry is a hard task, due to losses, of neutral species. However, the incorporation of 7-deaza purines reduces the neutral losses generating informative spectra. High mass accuracy in ESI–FT–ICR–MS combined with 7-deaza purines in the PCR can provide an integrated strategy for broad-based genotyping and is expected to have a major impact in human genetics, informative and forensic science.

Although MALDI processes are still poorly understood, many efforts are being made to combine high mass accuracy and simplicity of data interpretation with the ability to obtain extra structural information from reliable MS–MS data.

In his presentation entitled '**Applications of the MALDI-QqTOF Mass Spectrometer: Its High Sensitivity, High Resolution, High Mass Accuracy and High Throughput Make It a Powerful Tool for Protein Identification'**, Kenneth G. Standing (University of Manitoba, Canada) showed data in which the MALDI-QqTOF was used to perform typical proteomics experiments that allow easy protein identification. This is a new mass spectrometer in which a MALDI ion source has been interfaced to an orthogonal injection time-of-flight analyser, allowing tandem mass spectrometry with a MALDI instrument similar to the equivalent ESI mass spectrometer, in which a precursor ion can be selected in the quadrupole Q and fragmented in the collision cell q (Shevchenko *et al.*, 2000). Measurements of the product ions in the TOF section provides an MS–MS spectrum of the selected precursor. Identification of proteins separated by one or two-dimensional gel electrophoresis at the femtomole level have been reported; the acquisition of MS and MS–MS spectra with database searching tools lead to an unambiguous identification of complex protein mixtures. Proteins can be readily identified in all types of sequence databases including EST.

In a MALDI TOF-TOF system, the standard MALDI-TOF is modified by adding another TOF analyser, so that tandem mass spectrometry is carried out using a MALDI source. The ions leaving the first time-of-flight analyser are selected to be fragmented by collision induced dissociation (CID) and the fragments are subsequently measured using the second TOF analyser (Medzihradszky *et al.*, 2000). The TOF-TOF MALDI mass spectrometer displays an extended higher energy fragmentation with respect to the MALDI-qQTOF mentioned previously.

Marvin Vestal, from PE-Biosystems (now renamed Applied Biosystems, Framingham, MA, USA), presented a talk entitled '**Performance Evaluation of Improved MALDI TOF-TOF MS System'** The parameters studied included resolution, mass accuracy, sensitivity and dynamic range in both MS and MS–MS modes. Particular emphasis was placed on the attempt to determine absolute ionization and transmission efficiency, and on evaluating the effects of collision cell pressure and collision energy on fragmentation spectra. The new prototype has a resolution greater than 15 000 (within the range typically covered by a protein digest, ca. 600–4000 $m/z$) and mass accuracy with internal calibration is lower than 10 ppm for all peaks with relative abundances greater than 1%.

Curiously, both new MALDI mass spectrometers are being developed by the same company, generating an internal competition between two items that should attract the same clientele.

In '**Activated Ion Electron Capture Dissociation of Large Biomolecules'**, presented by David Horn (Cornell University, Ithaca, NY, USA), electron capture dissociation (ECD) was shown to provide substantially more sequence information than collisionally-activated decomposition (CAD) for proteins with molecular weights up to 12 kDa. However, larger proteins ($>17$ kDa) show very little fragmentation, due to the strong gas-phase conformational stability of these larger biomolecules. Different forms of activation prior to protein denaturation were reported. This is particularly important to enable ECD of large proteins; blackbody infra-red radiative dissociation (BIRD) was successfully applied to increase the amount of sequence information for cytochrome *c* $9+$ and $15+$ molecular ions and for monitoring their gas phase conformation. Different fragmentation patterns at different temperatures were apparent in the ECD experiments for both cytochrome *c* $9+$ and $15+$, indicating the unfolding of the protein.

In the same session, Lori Smith (University of Arizona, Tucson, AZ, USA) presented her work,

Copyright © 2000 John Wiley & Sons, Ltd. 

giving a talk entitled **'SID vs. CID: a Comparison of Peptide Sequencing Information Content of MS–MS Spectra'**, in which surface-induced dissociation (SID) was compared to collision-induced dissociation (CID) to evaluate SID as a complementary activation technique for protein identification.

SID has been established as an excellent tool for studying mechanisms of fragmentation of protonated peptides; collision between the selected ion and the surface enables the conversion of a larger amount of kinetic energy into internal energy, resulting in extensive fragmentation. Typical SID fragmentation patterns of tryptic digest peptides are different with respect to spectra produced under CID conditions. Neverthless, the most abundant fragments generated under SID can be exploited for protein identification. Analysis of small arginine and lysine terminal peptides using SID has been reported: such peptides have been chosen to mimic tryptic digest products. The additional information from SID spectra reduces the number of peptide sequence candidates and aids the production of correct sequences from MS–Tag. Collision-induced dissociation (CID) also generates fragmentation data, but the probability of obtaining false positives is high if none of the amino acid sequence is known. Thus, SID activation is a complementary activation technique that increases the level of information obtained from MS–MS analysis.

Although SID spectra contain more ions that could be exploited for protein identification, one drawback of this activation technique is that it necessitates a large amount of analyte, which is rarely available in biological samples.

Although MALDI processes remain poorly understood, scientists are developing microanalysis techniques that allow the operator to handle thousands of samples every day. In the field of MS chip technology, a **2D/3D chip for MALDI-MS** has been presented by Eckhard Nordhoff (Max-Planck-Institute for Molecular Genetics, Berlin, Germany). A prestructured MALDI–MS sample support has been developed to simplify high-throughput analysis of biomolecules (Schüerenberg *et al.*, 2000). The 2D/3D-chip design enables chemical reactions to be performed on a small number of biomolecules with minimal losses caused by surface adsorption. The planar support is equipped with a thin layer of hydrophobic Teflon that carries an array of 2000 μm gold spots, which provide hydrophilic sample anchors. Each transferred sample droplet is loaded onto the anchor, on top of which, after solvent evaporation, the sample is retained due to the strongly water-repellent nature of the Teflon surface. Using this support, detection sensitivity is improved up to the attomole level.

### Poster sessions

In **'Derivatization and Postsource Decay MALDI Mass Spectrometry for Definitive Protein Identification in Proteomics Research'**, Thomas Keough (Procter & Gamble Co., Cincinnati, OH, USA) has presented new results about his method for *de novo* peptide sequencing (Keough *et al.*, 1999). An optimized procedure has been developed for the addition of the sulphonic acid group to the N-termini of tryptic digest peptides. The method has been applied to arginine-containing peptides generated by tryptic digestion of proteins separated by 2D gel electrophoresis. In MALDI, singly charged ions are mostly generated during the ionization process. Although the metastable decay occurring after the ions leave the MALDI source can be monitored by using post-source decay (PSD), the intrinsic features of tryptic digest peptides that contain basic C-terminal amino acids (arginine and lysine) do not allow effective fragmentation. If the N-terminus is modified by derivatization with a negative sulphonic acid group, the sulphonyl-containing peptide must contain two positive charges that are detected as a singly charged species. Hence, whereas one charge is sequestered by the basic amino acid, the second proton is mobile, enabling fragmentation that leads exclusively to the formation of C-terminal fragments.

In a second poster, the application of nanoelectrospray ionization tandem mass spectrometry (nES–MS–MS) and capillary LC/microelectrospray MS–MS (cLS/nES–MS–MS) for sequencing sulphonic acid-derivatized tryptic peptides was reported by the same author. The content of the poster has already been published in *Rapid Comm Mass Spectrom* (Bauer *et al.*, 2000).

In the poster entitled **'In-source Decay (ISD) Characteristics of Peptide and Protein in MALDI-TOF–MS Compared to ISD and CID'**, Matzuo-Takayama (Toho University, Funabashi, Japan) presented in-source-decay MALDI applied to several polypeptides and proteins. For rapid identification, ISD can provide appropriate internal sequence

Copyright © 2000 John Wiley & Sons, Ltd.

information without pre-digestion of proteins. CID and PSD, however, necessitate prior hydrolysis of proteins with trypsin to produce peptide mixtures whose fragments are smaller than ca. 3000 Da. A striking advantage of ISD is the appearance of relatively specific and simple products such as c-, y- and/or z-series, that could be essential in reconstructing the protein sequence.

A new MALDI probe has been developed and was presented by Ryan M. Danell (University of North Carolina, Chapel Hill, NC, USA) in the poster entitled '**An Atmospheric Pressure MALDI Probe for Use with ESI Source Interfaces'.** This new probe has been demonstrated to operate either at atmospheric pressure (AP) or under standard vacuum conditions. The probe is easily interfaced with capillary electrospray ionization sources, enabling the same instrument to record both ESI and MALDI spectra with little variation. The new probe is made of a thin-walled glass capillary tube into which crystals are deposited. A fibre-optic is used to illuminate the target with the laser. The design of the MALDI probe enables easy implementation with direct-insertion probe interfaces and the easy interchange with capillary-based ESI sources provides a useful addition for many mass spectrometers.

A new ionization strategy for biomolecular mass spectrometry has been developed by Gary Siuzdak (Scripps Research Institute, La Jolla, CA, USA), based on pulsed laser desorption/ionization from a porous silicon surface (Wei *et al.*, 1999). Two posters were presented on the desorption ionization on silicon (DIOS), whose titles were '**Aspects of Desorption/Ionization on Porous Silicon (DIOS) Mass Spectrometry**', presented by Zhouxin Shen (Scripps Research Institute, La Jolla) and '**Desorption/Ionization on Porous Silicon (DIOS) for Proteomics**', presented by John Thomas (Scripps Research Institute, La Jolla). Desorption/ionization on silicon (DIOS) uses porous silicon to trap analytes deposited on the surface, and laser radiation to vaporize and ionize these molecules. The main advantage lies in the ability to perform MALDI analysis, generating spectra with no interfering signals due to matrix. When more evidence and details about preparation of the support and applicability to different analytes become available, such an ionization technique could well be worthwhile for scientists working on drug metabolism and making analyses of low-molecular weight compounds.

A new strategy to facilitate the detection of tryptic fragments and protein identification was presented in the poster entitled **'A Combination of Chemical Derivatization and Improved Bioinformatic Tools Optimizes Protein Identification for Proteomics'**, by Francesco Brancia (UMIST, Manchester, UK). In this procedure, peptide fragments are subjected to two forms of derivatisation (Brancia *et al.*, in press). First, lysine residues are converted to homoarginine moieties by guanidination. This procedure has two advantages; it usually identifies the C-terminal amino acid of the tryptic peptide and also greatly increases the total information content of the mass spectrum by improving the signal response of C-terminal lysine fragments. Second, an Edman-type phenylthiocarbamoyl (PTC) modification is carried out, which renders the first peptide bond highly susceptible to cleavage during MS analysis and consequently allows the ready identification of the N-terminal residue. The utility of these procedures has been demonstrated by developing novel bioinformatic tools to exploit the additional mass spectral data in the identification of proteome proteins from the yeast *Saccharomyces cerevisiae.* New software tools, **PepMAPPER**, are freely available on the Internet (**http://wolf.bi.umist.ac.uk/mapper**). With this combination of novel chemistry and bioinformatics, it should be possible to identify unambiguously any yeast protein spot or band from either two-dimensional or one-dimensional electrophoretograms.

### References

Bauer MD, Sun YP, Keough T, Lacey MP. 2000. Sequencing of sulfonic acid derivatized peptides by electrospray mass spectrometry. *Rapid Comm Mass Spectrom* **14**: 924–929.

Brancia FL, Butt A, Beynon RJ, Hubbard SJ, Gaskell SJ, Oliver SG. A combination of chemical derivatisation and improved bioinformatic tools optimizes protein identification for proteomics. *Electrophoresis* (in press).

Keough T, Youngquist RS, Lacey MP. 1999. A method for high-sensitivity peptide sequencing using post-source decay matrix-assisted laser desorption ionization mass spectrometry. *Proc Natl Acad Sci U S A* **96**: 7131–7136.

Medzihradszky KF, *et al.* 2000. The characteristics of peptide collision-induced dissociation using a high-performance MALDI-TOF/TOF tandem mass spectrometer. *Anal Chem* **72**: 552–558.

Schüerenberg RM, Luebbert C, Eickhoff H, Kalkum M, Lehrach

H, Nordhoff E. 2000. Prestructured MALDI–MS sample supports. *Anal Chem* **72**: 3436–3442.

Shevchenko A, Loboda A, Shevchenko A, Ens W, Standing KG. 2000. MALDI quadrupole time-of-flight mass spectrometry: a powerful tool for proteomic research. *Anal Chem* **72**: 2132–2141.

Wei J, Burlak JM, Siuzdak G. 1999. Desorption–ionization mass spectrometry on porous silicon. *Nature* **399**: 243–246.

*The*
**Genomics**
*Website at Wiley*

**www.wiley.co.uk/genomics**

The **Genomics** website at Wiley is a new and **DYNAMIC** resource for the genomics community, offering **FREE** special feature articles and new information **EACH MONTH**.

Find out more about *Comparative and Functional Genomics*, and how to view all articles published this year **FREE OF CHARGE**!

Visit the **Library** for hot books in Genomics, Bioinformatics, Molecular Genetics and more.

Click on **Primary Research** for information on all our up-to-the minute journals, including: *Genesis*, *Bioessays, Gene Function and Disease*, and the *Journal of Gene Medicine.*

Let the **Genomics** website at Wiley be your **guide** to genomics-related web sites, manufacturers and suppliers, and a calendar of conferences.

WILEY

1516

Copyright © 2000 John Wiley & Sons, Ltd. 
